# Incidence of acute otitis media in children < 16 years old in Germany during 2014–2019

**DOI:** 10.1186/s12887-022-03270-w

**Published:** 2022-04-13

**Authors:** Tianyan Hu, Bélène Podmore, Rosemarie Barnett, Dominik Beier, Wolfgang Galetzka, Nawab Qizilbash, Dennis Haeckl, Jessica Weaver, Timo Boellinger, Sarah Mihm, Tanaz Petigara

**Affiliations:** 1 Merck & Co., Inc, Kenilworth, NJ USA; 2 OXON Epidemiology, London, UK; 3 London School of Hygiene & Tropical Medicine, London, UK; 4 University of Bath, Bath, UK; 5 InGef – Institute for Applied Health Research Berlin GmbH, Berlin, Germany; 6 WIG2 GmbH, Leipzig, Germany; 7 MSD Sharp & Dohme GmbH, Haar, Germany

**Keywords:** Acute otitis media, Recurrent AOM, Complications, Surgical procedures, Pneumococcal conjugate vaccine, Healthcare claims

## Abstract

**Background:**

Acute otitis media (AOM) remains a common infection in children despite the introduction of pneumococcal conjugate vaccines. This study estimated AOM incidence rates (IRs) over time in children < 16 years old in Germany following PCV13 introduction.

**Methods:**

AOM episodes were identified in the InGef healthcare claims database from 2014–2019 in children aged < 16 years. Each AOM episode was classified as either simple or recurrent. Recurrent AOM was defined as 3 or more episodes identified within a 6-month period; or 4 or more episodes within a 12-month period with at least one episode in the prior 6 months. AOM-related surgical procedures within 12 months and complications within 21 days of an AOM episode were also identified. Annual IRs were calculated as number of episodes/child-years (CY) at risk. 95% Confidence intervals (95%CI) were calculated using the Wilson method. The Mann–Kendall test was used to assess trends over time.

**Results:**

Between 2014 and 2019, the study population comprised 916,805 children with 327,726 AOM episodes, of which 15% (49,011) of all episodes were identified as recurrent AOM and 85% (278,715) as simple AOM. There were significant declines in AOM (*p* = 0.003) in the study population overall and in all age groups over the study period; from 101 (95%CI 101–102)/1000 CY to 79 (95%CI 78–80)/1000 CY in the total study population, from 209 (95%CI 206–212)/1000 CY to 147 (95%CI 145–150)/1000 CY in < 2-year-olds, from 239 (95%CI 237–242) to 179 (95%CI 177–182)/1000 CY in 2–4-year-olds, and from 50 (95%CI 49–50) to 38 (95%CI 37–39)/1000 CY in 5–15-year-olds. No significant trends were identified for AOM-related surgical procedures over the study period; however, AOM-related complications overall increased (*p* = 0.003).

**Conclusion:**

Between 2014 and 2019, AOM incidence overall declined in children aged 0–15 years in Germany. Over the study period, the incidence of complicated AOM cases increased, however the incidence of AOM-related surgical procedures remained constant. Despite the impact of PCV13, the burden associated with AOM in Germany remains substantial.

**Supplementary Information:**

The online version contains supplementary material available at 10.1186/s12887-022-03270-w.

## Background

Acute otitis media (AOM) is a common childhood infection characterised by middle ear effusion, inflammation of the tympanic cavity and the rapid onset of symptoms and signs of an acute inflammation [1]. Approximately 75% of non-PCV immunized children are estimated to have at least one AOM episode before the age of 5 [2]. By the age of two years, up to 5% of children have experienced recurrent AOM, defined as three or more AOM episodes in six months or four or more episodes in one year [3]. AOM is a leading cause of office visits and antibiotic prescriptions in children [4–7]. Healthcare resource utilization associated with the management of AOM and AOM-related surgical procedures is substantial [8–10]. Although rare, complications of AOM such as acute mastoiditis and meningitis are associated with significant morbidity [11–13].

*Streptococcus pneumoniae (S. pneumoniae)* is a common bacterial cause of AOM [14, 15]. The incidence of AOM has declined in Europe and globally since the introduction of pneumococcal conjugate vaccines (PCVs) [16–20]. In Germany, universal vaccination of children aged < 2 years with the 7-valent PCV (PCV7) was recommended in 2006 and was replaced by 10- and 13-valent PCVs in 2009 (PCV10 and PCV13) [21]. In Germany vaccinations are voluntary, although strongly advised by health authorities. Statutory health insurance (SHIs) providers pay for vaccinations recommended by the German standing committee on vaccination (STIKO) and in alignment with the official vaccination guidelines published by the Robert Koch Institute [22]. Vaccinations are therefore free of charge via individuals’ SHI package. Public opinions on vaccination are generally positive [23]. The schedule for PCV vaccination in children is at two, four and 11–14 months old.

To better understand the potential value of new vaccines in Germany, it is important to quantify the current incidence of AOM. However, post-PCV13 data on AOM is lacking. In the present study, we therefore aimed to provide recent estimates for the incidence of all-cause AOM (overall, simple, and recurrent), AOM-related surgical procedures and AOM-related complications over time in children < 16 years old in Germany.

## Methods

### Data source

This is a retrospective cohort study of children aged < 16 years in Germany from January 1, 2014 to December 31, 2019 within the InGef (Institute for Applied Health Research Berlin GmbH, formerly Health Risk Institute) research database.

In Germany, approximately 85% of the population are insured by SHI providers; the remaining population insured by private health insurers. For research purposes, data can be pooled from multiple SHIs [24]. The InGef database is comprised of de-identified longitudinal claims data from approximately 7.2 million individuals across approximately 60 SHIs throughout all geographic regions in Germany. The sample dataset of approximately 4 million individuals used for the present study covers around 5% of the German population and is nationally representative in terms of age and sex [25]. Individuals can be followed over a longitudinal period of up to a maximum of six years due to data protection regulations. The database includes demographic information (gender, age, region of residence); diagnoses; claims data for ambulatory services and procedures in alignment with the German uniform evaluation standard (EBM,‘Einheitlicher Bewertungsmaßstab’); data on outpatient visits and inpatient hospital admissions (including admission and discharge dates, both primary and secondary discharge diagnoses and codes for procedures conducted in hospital according to the German Procedure Classification (OPS,‘Operationen und Prozedurenschlüssel‘)); mortality and morbidity data; drug prescription and dispensing data (drug class and identification number of each package (PZN, ‘Pharmazentralnummer’), quantity, date of prescription and dispensing); and healthcare costs [25]. All diagnoses are recorded using the German modification of the 10th revision of the International Classification of Diseases (ICD-10-GM).

### Study design

The study population comprised children aged < 16 years in Germany with statutory health insurance, within the InGef research database. Multiple yearly cohorts were created to assess the incidence of AOM within each calendar year of the study. Six yearly cohorts were established (2014 to 2019), whereby children contributed data from the latest of the following dates: start of the study year (January 1), estimated date of birth, or the date their SHI provider started contributing data to the InGef research database. For children who were born before 2014, their study entry date was assigned to the latter of January 1, 2014 or the date their SHI provider started contributing data to the InGef research database within the study period. During each study year, each individual was followed-up from the latest of the aforementioned dates until the first of the following censoring criteria: end of observation in the InGef research database (based on: end of insurance with SHI contributing data to the InGef research database, death from any cause), end of study year (December 31 of each year), or end of study period (December 31, 2019).

### Outcomes

AOM was defined as acute suppurative and unspecified otitis media caused by all known and unknown pathogens. AOM was identified in the InGef research database through ICD-10-GM codes within both the outpatient data and inpatient data (all diagnosis fields). As diagnoses are only available by quarter in the database for outpatient visits, at least one antibiotic prescription or diagnostic test in the same quarter was required to accompany an outpatient diagnosis of AOM. Within each quarter of an AOM diagnosis, the date of first antibiotic or diagnostic test was assigned as the date of diagnosis. Date of hospital admission was used to identify diagnosis dates within the inpatient data. Exact code definitions are outlined in the supplementary material, including EBM and OPS codes used to approximate outpatient diagnosis dates.

Multiple records were considered as independent episodes if they were ≥ 14 days apart [26]. Since analyses were conducted by calendar year, episodes were assigned to each study year. Episodes that crossed calendar years were assigned to the year in which the episode began. In alignment with the widely accepted definition of recurrent AOM (first coined by Goycoolea in 1991 [27]), each AOM episode was defined as simple or recurrent, whereby an individual was considered to have recurrent AOM if they had three or more episodes within a 6-month period or 4 or more episodes within a 12-month period after the first AOM episode, with at least one episode in the preceding six months. To avoid misclassification of a recurrent episode, only children with 6 months of continuous health plan enrolment prior to the first episode and 12 months of continuous health plan enrolment after the first episode were included. The 6-month period prior to the first episode was not required for children < 1 year of age.

AOM-related surgical interventions (myringotomy (with or without insertion of a tympanic drainage), tympanostomy tube removal, exploratory tympanotomy, tympanotomy with sealing of the round and/ or oval window membrane [28–30]) were identified through OPS codes and could occur anytime within 12 months after the AOM episode in children with at least one AOM episode. AOM-related complications (perforation of tympanic membrane, otorrhea, otorrhagia, acute mastoiditis) were identified through ICD-10-GM codes in both the outpatient and inpatient data (all diagnosis fields) in children with at least one AOM episode. AOM-related complications could occur anytime within 21 days of the start date of each AOM episode [26].

### Statistical methods

All analyses were completed using the statistical software program R, version 3.5.0. The study population was described by age (< 2 years, 2–4 years, and 5–15 years), sex (male, female), region (East, West, Berlin) and underlying medical conditions associated with an increased risk of pneumococcal disease according to the 2017/2018 STIKO recommendations for at-risk/high-risk individuals [31]. Underlying medical conditions were assessed in a 12-month look-back period for each individual from the date of study entry. As no medical history was available prior to 2014, underlying medical conditions were reported only for 2015–2019. The underlying medical conditions were identified by ICD-10-GM codes in the outpatient and inpatient data (all diagnosis fields).

AOM incidence rates (IRs) per 1,000 child-years (CY) were calculated as the number of AOM episodes divided by the sum of CY at risk, whereby time at-risk was defined as the total follow-up time minus the time with AOM. 95% Confidence intervals (95% CI) were calculated using the Wilson method [32]. IRs were estimated for AOM overall, as well as simple AOM, and recurrent AOM in each study year. IRs of AOM-related surgical procedures per 1,000 CY were calculated as the number of procedures divided by sum of CY at risk also, whereby time at risk was classified as within 12 months following each AOM episode. Similarly, IRs of AOM-related complications were calculated as the number of complications divided by the sum of CY at risk, with time at risk classified as within 12 months following each AOM episode [26]. IRs of AOM, simple AOM, recurrent AOM, AOM-related surgical procedures and AOM-related complications were further stratified by age groups (< 2 years, 2–4 years, and 5–15 years). To assess whether the incidence changed significantly between 2014 and 2019, Mann–Kendall linear trend tests were used.

## Results

The study population included 916,805 children aged < 16 years. These individuals contributed a total of 3,608,716 CY at-risk and were followed up for a median of 4.3 (interquartile range 2.2–6) years. At annual cohort entry, the mean age of individuals was 6 years (standard deviation 5.2). The majority of children were 5–15 years old at cohort entry (52.7%) (Table 1) and from the Western region of Germany (82.4%). For each yearly cohort, near 90% of individuals had no history of any underlying medical condition associated with pneumococcal disease (range: 89.2% in 2015 cohort to 91.8% in the 2019 cohort). The most common comorbidity was chronic pulmonary disease, including asthma (ranging from 5.2% in the 2019 cohort to 7.1% in the 2015 cohort).

**Table 1:** Baseline characteristics of the study population from 2014–2019 (*n* = 916,805)

Variable	N	%
**Population by year**		
2014	683,807	-
2015	682,618	-
2016	673,787	-
2017	662,069	-
2018	654,311	-
2019	646,472	-
**Age (years) at study entry**		
Mean, standard deviation	6	5.22
**Age group (n, %)**		
0–1	306,767	33.46
2–4	126,764	13.83
5–15	483,274	52.71
**Sex (n, %)**		
Male	471,991	51.48
Female	444,814	48.52
**Geographic region (n, %)**		
East	92,474	10.09
West	755,537	82.41
Berlin	67,173	7.33
Unknown	1,621	0.18
**Underlying medical conditions for 2015 cohort**^**a**^** (n, %)**		
No at-risk medical condition	609,198	89.24
Any at-risk medical condition	67,001	9.82
Chronic diseases		
* Insulin-dependent diabetes mellitus*	2,089	0.31
* Chronic pulmonary disease (incl. asthma)*	48,728	7.14
* Chronic heart disease*	11,535	1.69
* Neurological disorders*	8,195	1.20
Any high-risk medical condition	9,359	1.37
* Cancer*	931	0.14
* Cerebrospinal fluid leak*	8	0.00
* Chronic renal disease*	848	0.12
* Cochlear implant*	1,078	0.16
* Functional or anatomic asplenia, sickle cell disease/other hemaglobinopathy, congenital or acquired asplenia, splenic dysfunction, splenectomy*	737	0.11
* HIV infection*	27	0.00
* Immuno-compromising diseases*	5,287	0.77
* Organ transplant*	431	0.06
* Chronic liver disease*	408	0.06
* Autoimmune disease*	368	0.05

^a^Underlying medical conditions were assessed in a 12-month look-back period for each individual from the date of study entry. As no medical history was available prior to 2014, results are displayed for the 2015 cohort

### Incidence of AOM

Overall IRs and for each age group are presented in Table 2. From 2014–2019, there were 327,726 episodes of AOM in total; the average annual IR for the study period was 91.1 (95%CI 90.8–91.5) episodes per 1,000 CY. Annual IRs of AOM declined significantly over the study period, from 101.4 (95%CI 100.6–102.2) in 2014 to 79.1 (95%CI 78.4–79.8) per 1,000 CY in 2019 (trend test, *p* = 0.003). When stratified by age, AOM IRs declined from 209.0 (95%CI 205.6–212.5) to 147.5 (95%CI 144.7–150.3) per 1,000 CY in children < 2 years, from 239.4 (95%CI 236.6–242.2) to 179.4 (95%CI 177.0–181.8) per 1,000 CY in children 2–4 years, and from 49.8 (95%CI 49.2–50.5) to 37.9 (95%CI 37.3–38.5) per 1,000 CY in children 5–15 years. Mann–Kendall tests indicated that the decline in IRs overall, and for each age group, were all significant.

**Table 2:** Incidence rates of AOM in the overall study population, over the total study period by age group

	**Overall (2014–2019)**	**2014**	**2015**	**2016**	**2017**	**2018**	**2019**	**Trend test (p-value) **^**a**^
**Overall (all age-groups)**								
* N episodes*	327,726	64,045	61,289	59,113	52,840	50,242	47,346	
* Rate per 1,000 CY (95% CI)*	91.14 (90.83–91.45)	101.36 (100.58–102.15)	97.57 (96.80–98.35)	95.91 (95.14–96.69)	86.71 (85.97–87.45)	83.06 (82.34–83.79)	79.11 (78.40–79.83)	0.003
**0–1 age group**								
* N episodes*	75,400	14,442	13,667	13,293	12,137	11,465	10,765	
* Rate per 1,000 CY (95% CI)*	174.55 (173.31–175.80)	209.01 (205.62–212.45)	191.76 (188.56–195.01)	182.80 (179.71–185.93)	164.76 (161.84–167.72)	155.65 (152.82–158.53)	147.49 (144.71–150.30)	0.003
**2–4 age group**								
* N episodes*	139,403	27,225	25,607	24,862	22,728	21,535	21,112	
* Rate per 1,000 CY (95% CI)*	207.65 (206.56–208.74)	239.38 (236.55–242.24)	224.05 (221.31–226.81)	217.55 (214.85–220.27)	196.75 (194.20–199.32)	183.22 (180.78–185.68)	179.39 (176.98–181.83)	0.003
**5–15 age group**								
* N episodes*	112,923	22,378	22,015	20,958	17,975	17,242	15,469	
* Rate per 1,000 CY (95% CI)*	45.30 (45.04–45.57)	49.84 (49.19–50.49)	49.74 (49.09–50.41)	48.82 (48.16–49.48)	42.78 (42.15–43.41)	41.68 (41.06–42.31)	37.94 (37.34–38.54)	0.003

^a^ Mann–Kendall test for trend

IRs were greatest in children aged 2–4 years (207.7 (95%CI 206.6–208.7) per 1,000 CY), followed by children < 2 years (174.6 (95%CI 173.3–175.8) per 1,000 CY), and 5–15 years (45.3 (95%CI 45.0–45.6) per 1,000 CY).

### Incidence of simple versus recurrent AOM

The IRs of simple and recurrent AOM overall and for each age group during the study period are presented in Tables 3 and 4. Mann–Kendall tests indicated that for all children and in each age group, there were significant monotonic declines for IRs of simple AOM and recurrent AOM over the study period. The average annual IR for simple AOM (77.5 (95%CI 77.2–77.8) per 1,000 CY) was significantly higher than for recurrent AOM (13.6 (95%CI 13.5–13.7) per 1,000 CY) over the study period. The IRs of simple AOM were greatest in children 2–4 years (168.9 (95%CI 167.9–169.9) per 1,000 CY), followed by those in children < 2 years (143.8 (95%CI 142.7–145.0) per 1,000 CY) and 5–15 years (41.3 (95%CI 41.1–41.6) per 1,000 CY). Similarly, IRs of recurrent AOM were highest in children 2–4 years, followed by those in children < 2 years and 5–15 years.

**Table 3:** Incidence rates of simple AOM in the overall study population over the total study period by age group

	**Overall (2014–2019)**	**2014**	**2015**	**2016**	**2017**	**2018**	**2019**	**Trend test (*p*-value)**^**a**^
**Overall (all age-groups)**								
* N episodes*	278,715	53,306	51,507	50,079	45,118	43,414	41,528	
* Rate per 1,000 CY (95% CI)*	77.47 (77.18–77.76)	84.31 (83.59–85.03)	81.95 (81.24–82.66)	81.21 (80.50–81.92)	74.00 (73.32–74.69)	71.74 (71.07–72.42)	69.36 (68.70–70.04)	0.003
**0–1 age group**								
* N episodes*	62,198	11,544	11,034	10,950	10,109	9,704	9,137	
* Rate per 1,000 CY (95% CI)*	143.82 (142.69–144.95)	166.79 (163.76–169.86)	154.59 (151.72–157.50)	150.39 (147.59–153.23)	137.08 (134.42–139.78)	131.62 (129.01–134.26)	125.07 (122.52–127.66)	0.003
**2–4 age group**								
* N episodes*	113,561	21,612	20,471	20,083	18,618	17,860	17,980	
* Rate per 1,000 CY (95% CI)*	168.89 (167.91–169.88)	189.65 (187.13–192.19)	178.79 (176.35–181.25)	175.44 (173.02–177.88)	160.94 (158.63–163.27)	151.76 (149.55–154.01)	152.61 (150.39–154.86)	0.02
**5–15 age group**								
* N episodes*	102,956	20,150	20,002	19,046	16,391	15,850	14,411	
* Rate per 1,000 CY (95% CI)*	41.30 (41.05–41.55)	44.87 (44.25–45.49)	45.19 (44.56–45.82)	44.35 (43.73–44.99)	39.00 (38.41–39.60)	38.31 (37.71–38.91)	35.34 (34.76–35.92)	0.02

^a^ Mann–Kendall test for trend

**Table 4:** Incidence rates of recurrent AOM in the overall study population over the total study period by age group

	**Overall (2014–2019)**	**2014**	**2015**	**2016**	**2017**	**2018**	**2019**	**Trend test (*p*-value) **^**a**^
**Overall (all age-groups)**								
* N episodes*	49,011	10,739	9,782	9,034	7,722	6,828	5,818	
* Rate per 1,000 CY (95% CI)*	13.59 (13.47–13.71)	16.94 (16.62–17.26)	15.52 (15.22–15.83)	14.61 (14.31–14.92)	12.64 (12.35–12.92)	11.26 (10.99–11.53)	9.70 (9.45–9.95)	0.003
**0–1 age group**								
* N episodes*	13,202	2,898	2,633	2,343	2,028	1,761	1,628	
* Rate per 1,000 CY (95% CI)*	30.39 (29.88–30.92)	41.67 (40.17–43.22)	36.72 (35.33–38.15)	32.03 (30.75–33.36)	27.39 (26.21–28.60)	23.79 (22.69–24.92)	22.20 (21.13–23.30)	0.003
**2–4 age group**								
* N episodes*	25,842	5,613	5,136	4,779	4,110	3,675	3,132	
* Rate per 1,000 CY (95% CI)*	38.24 (37.77–38.71)	48.99 (47.71–50.29)	44.62 (43.41–45.86)	41.53 (40.36–42.73)	35.36 (34.28–36.45)	31.08 (30.08–32.10)	26.45 (25.54–27.40)	0.003
**5–15 age group**								
* N episodes*	9,967	2,228	2,013	1,912	1,584	1,392	1,058	
* Rate per 1,000 CY (95% CI)*	3.99 (3.91–4.07)	4.95 (4.75–5.16)	4.54 (4.34–4.74)	4.45 (4.25–4.65)	3.76 (3.58–3.95)	3.36 (3.19–3.54)	2.59 (2.44–2.75)	0.003

^a^ Mann–Kendall test for trend

### Incidence of AOM-related surgical procedures

**Fig. 1:**
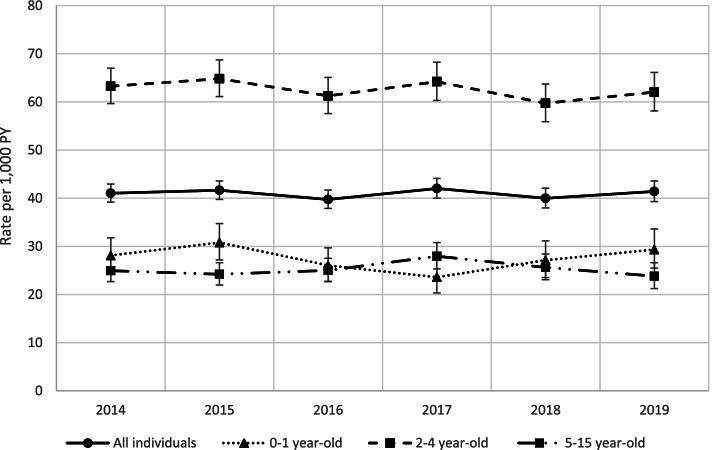
Rate of AOM-related surgical procedures over the study period (2014–2019) by age group

Over the study period there were a total of 9,677 AOM-related surgical procedures, occurring within 12 months following the AOM index episode. The average annual IR of AOM-related surgical procedures over the total study period was 40.9 (95%CI 40.1–41.7) per 1,000 CY. The rate of AOM-related surgical procedures remained steady over the study period: Mann–Kendall tests indicated no significant trends overall or by age group (Fig. 1, Supplementary Table 5). Rates were consistently highest in the 2–4-year-old age group over the study period: 27.4 (95%CI 25.9–28.9), 62.7 (95%CI 61.2–64.3), and 25.0 (95%CI 24.0–26.1) per 1,000 CY for children < 2, 2–4 and 5–15 years, respectively (Fig. 1).

### Incidence of AOM-related complications

There were 10,385 AOM-related complications over the study period, occurring within 21 days of an AOM episode (Table 5). Average annual IR of AOM-related complications was 43.9 (95%CI 43.0–44.7) episodes per 1,000 CY. The Mann–Kendall test indicated a significant increase in the rate of AOM-related complications over the study period, overall, for the < 2 age group and for the 5–15 age group. Rates were similar across all age groups: 45.8 (95%CI 43.9–47.8), 44.0 (95%CI 42.7–45.3), and 42.7 (95%CI 41.4–44.1) per 1,000 CY for children < 2, 2–4 and 5–15 years, respectively.

**Table 5:** Incidence rates of AOM-related complications occurring within 21 days of the start of an AOM episode

	**Overall (2014–2019)**	**2014**	**2015**	**2016**	**2017**	**2018**	**2019**	**Trend test (*p*-value)**^**a**^
**Overall (all age-groups)**								
* N episodes*	10,385	1,712	1,781	1,887	1,763	1,711	1,772	
* Rate per 1,000 CY (95% CI)*	43.86 (43.02–44.71)	37.83 (36.06–39.66)	40.71 (38.84–42.64)	44.37 (42.39–46.42)	45.89 (43.78–48.09)	46.17 (44.01–48.42)	50.49 (48.17–52.90)	0.003
**0–1 age group**								
* N episodes*	2,213	375	334	358	367	368	422	
* Rate per 1,000 CY (95% CI)*	45.81 (43.92–47.76)	41.50 (37.40–45.92)	38.79 (34.74–43.19)	41.79 (37.57–46.35)	46.55 (41.91–51.57)	49.43 (44.51–54.75)	60.08 (54.49–66.10)	0.017
**2–4 age group**								
* N episodes*	4,245	691	708	786	754	686	741	
* Rate per 1,000 CY (95% CI)*	44.00 (42.69–45.34)	37.85 (35.08–40.78)	40.72 (37.78–43.84)	46.11 (42.95–49.45)	47.55 (44.21–51.06)	44.86 (41.57–48.35)	48.95 (45.49–52.61)	0.056
**5–15 age group**								
* N episodes*	3,927	646	739	743	642	657	609	
* Rate per 1,000 CY (95% CI)*	42.69 (41.37–44.05)	35.97 (33.24–38.85)	41.62 (38.67–44.73)	43.92 (40.82–47.20)	43.75 (40.43–47.27)	45.88 (42.44–49.53)	47.08 (43.42–50.97)	0.017

^a^ Mann–Kendall test for trend

## Discussion

Next generation, higher-valent pneumococcal vaccines are under development to further reduce the burden of pneumococcal disease, including AOM [33–35]. To better understand the potential value of new vaccines in Germany, the present study provides recent estimates of the incidence of AOM in the post-PCV-13 era. AOM overall, simple AOM and recurrent AOM declined significantly over the study period (2014–2019). AOM incidence (overall, simple and recurrent) was greatest in children 2–4 years, followed by incidence in children < 2 years, and 5–15 years. Although the overall incidence of AOM declined over the study period, the incidence of complications increased and the incidence of AOM-related surgical procedures remained constant. AOM-related surgical procedures were again highest in children aged 2–4 years. IRs of AOM-related complications were similar across age groups.

Variations in study design, timeframe, case definition and age-groups complicate comparisons of IRs between AOM studies. Nonetheless, our results are consistent with the only other study of AOM incidence in Germany. In a prospective cohort study conducted after the introduction of PCV7 (2008–2010) in Germany, the incidence of physician-diagnosed AOM was 311 and 218/ 1,000 CY in children 0–2 and 3–5 years respectively [2]. Our study, after the introduction of PCV13, found a further decline in AOM incidence in children aged < 2 years, from 209/ 1,000 CY in 2014 to 147/ 1,000 CY in 2019. This is unsurprising, as in 2010, PCV13-PCV7 serotypes accounted for 60% of pneumococcal isolates in children < 5 years with spontaneous otorrhea [36].

Several studies before and after the introduction of PCV7 have demonstrated the significant impact of PCV introduction on pneumococcal serotype distribution in Germany. Two bacterial etiology studies were conducted in children < 5 years with spontaneously draining AOM prior to (1995–2007) and following the introduction of PCV7 (2008–2011) [36, 37]. Serotypes included in PCV7 accounted for 60.3% of pneumococcal isolates in the pre-vaccination period compared to only 8.3% of isolates following PCV7 introduction. A third study showed that diagnosis rates for suppurative and non-suppurative otitis media declined by 19% and 26%, respectively, in the post-vaccination period (2007–2011) compared to the pre-vaccination period (2003–2011) [3]. Furthermore, a more recent follow-up study demonstrated a dramatic decrease (-86%) in cases of AOM caused by *S. pneumoniae* over the seven study years (2008–2014); with a total disappearance of PCV7 and PCV13 serotypes, except for serotype 3 [38].

Other studies have observed significant declines in AOM IRs in children following PCV introduction. A recent study in Israel demonstrated a downward trend of IRs of AOM during the post-PCV years in children aged < 9 years (August 2009–2018; *P* < 0.001) [39]. The largest decrease (21%) was observed in children aged < 1 year, from 807/ 1,000 children during the pre-PCV years to 640/ 1,000 during the post-PCV years (*P* < 0.001). Similar results have been observed in children aged ≤ 5 years in Sweden: with a 2.3% decrease in otitis media and AOM following PCV13 introduction [40].

In this study, we found AOM incidence to be highest in the 2–4-year-old age group. Our findings are supported by studies in Italy and Eastern Europe. A study among children aged 0–6 years utilising the Italian Pedianet database (2003–2007) found that AOM incidence was highest in the 3–4 year old age group (222/ 1,000 CY; CI 218–227) [17]. Similarly, among children aged < 6 years in five Eastern European countries, AOM incidence was highest in the 3–4 year age group (209/ 1,000 CY; CI 165–261) between June 2011 and January 2013 [41]. Potential explanations in Italy may be the age at which children begin school and family size. Differences in the age distribution in Italy compared with other countries (where incidence is highest among children aged 6–24 months [42–44]) is likely due to day-care attendance, which in Italy typically starts around 3 years of age. Similarly, in Germany, kindergarten is typically attended by children between 3 and 6 years old [45]. It is well established that children who attend day-care have higher AOM IRs than their non-attending counterparts [45]. The small size of the average Italian family (2.47 members) and German family (2.0 members) may also reduce the risk of infections for infants through exposure to other children.

This study provides the most recent estimates of AOM-related surgical procedures in Germany. The incidence of AOM-related surgical procedures (myringotomy, tympanostomy tube removal, exploratory tympanotomy, tympanotomy with sealing of the round and/ or oval window membrane) remained steady throughout the study period, despite a decrease in the overall rate of AOM. A similar study in the US (2001–2011) observed a downward trend in the incidence of myringotomy/ ventilation tube insertion immediately after PCV13 introduction [26]. Similar trends have been observed in Sweden, with a downward trend in tympanostomy tube placement and myringotomy procedures after PCV13, compared with pre-PCV cohorts [46]. However, our findings (2014–2019) may be explained by the increase in AOM-related complications. Other studies have also found an increasing burden of AOM-related complications in the post-PCV era [26, 47–51]. Shifts in AOM etiology or pneumococcal serotype distribution as a result of PCV introduction can impact the incidence of AOM (overall, simple, or recurrent) and AOM-related surgical procedures and complications to different degrees. We were unable to further evaluate these trends in the current study due to lack of pathogen or serotype information in the InGef database.

The main strength of this study is the precision of the estimates due to the large study population and representativeness of the InGef database (approximately 4 million insured members). Previous studies have demonstrated age, sex, morbidity, mortality and drug prescription/dispensation distributions to be similar in the InGef database and German population [25]; although representativeness in terms of other factors such as socioeconomic status have not been assessed.

There were several limitations to this study. First, exact diagnosis dates were not available in the outpatient data so quarterly diagnosis dates were used instead. Antibiotic prescriptions and diagnostic tests during the quarters with AOM diagnoses were assumed to be related to AOM to assign exact diagnosis dates. However, this may have still led to inaccurate identification of AOM diagnosis dates. Second, misclassification bias due to coding inaccuracies of AOM is possible. However, this potential bias would serve to underestimate the incidence of AOM and our findings are not out of line with other studies [2, 17, 36, 37]. Furthermore, ICD-9 or ICD-10 codes have been used to identify AOM in many prior studies assessing AOM incidence, including recent studies in Germany and the US [52, 53].

In addition, the use of antibiotics prescriptions or diagnostic tests to validate an AOM diagnosis, may have led to a further underestimation of the true incidence of AOM in Germany as not all AOM episodes may have been treated with antibiotics or involved a diagnostic test. In many countries, including Germany, AOM clinical guidelines call for watchful waiting and to refrain from prescribing antibiotics for all AOM episodes [54]. However, the most recent AOM clinical guidelines in Germany explicitly state that those patients with uncomplicated AOM can initially be treated purely symptomatically, only if they undergo clinical examination and otomicroscopy/otoscopy (one of the diagnostic tests in the present study) after 2 to 3 days [28]. Therefore, uncomplicated AOM cases not treated with antibiotics should still have been captured through the presence of an otoscopy code. Updated standardised guidelines for the diagnosis and treatment of AOM in Germany are currently in development by the Arbeitsgemeinschaft der wissenschaltlichen medizinischen Fachgesellschaften (AWMF), due for completion at the end of 2023 [55].

Similarly, the focus in the present study was on extracranial AOM-related complications, and intracranial complications such as meningitis were therefore not captured [56]. The AOM-related complication rate is therefore likely to be underestimated. However, of the 221,123 patients who had AOM, only 96 had meningitis anytime across the study period. Therefore, seeing as meningitis is a rare complication, the number of patients developing meningitis within 21 days of an AOM episode is likely to be negligible [26]. Furthermore, not all AOM-related surgical procedures, for example mastoidectomy or drainage of the subperiosteal mastoid abscess, may have been captured due to the limitation of the procedure codes available in the InGef database. However, while mastoidectomy was a common surgical intervention associated with AOM in the pre-antimicrobial era, it is now believed to be performed in fewer than 5 cases per 100,000 people with AOM [57]. Indeed, other similar published studies have not included mastoidectomy when looking at surgical procedures associated with AOM or reported such low rates that statistical analysis was fruitless [19, 31].

A further limitation is that the IRs estimated in this study were not adjusted for covariates such as sex or chronic diseases as the aim of this study was to describe the incidence of AOM and AOM-related complications/surgical procedures in different age groups. Results are therefore presented as crude rates and CIs.

Finally, information on viral or bacterial AOM, causative pathogen (e.g., *Streptococcus pneumoniae, Haemophilus influenzae*, *Moraxella catarrhalis*) and serotype distribution was not available. An understanding of prevalent and emerging pneumococcal serotypes will be critical when considering the development and introduction of novel vaccines to reduce residual disease burden.

## Conclusions

We present recent estimates of all-cause AOM incidence in Germany. The incidence of AOM overall declined in Germany in children < 16 years old between 2014 and 2019, following the introduction of PCV13. While the incidence of simple AOM and recurrent AOM declined, significant trends were not identified for incidence of AOM-related surgical procedures. Furthermore, incidence of AOM-related complications increased. Despite the impact of PCV13 introduction, there remains substantial, residual burden associated with AOM in Germany. The impact of future PCVs on AOM IRs will depend on the proportion of AOM caused by *S. pneumoniae* and serotypes covered by vaccines.

## Supplementary Information

**Additional file 1: Supplementary Table 1.** AOM and OME ICD-10-GM code definitions. **Supplementary Table 2.** EBM and OPS codes for estimation of diagnosis date. **Supplementary Table 3.** AOM-related complications code definitions. **Supplementary Table 4.** AOM-related surgical procedures code definitions. **Supplementary Table 5.** Incidence rates of AOM-related surgical procedures occurring during the same calendar year as an AOM episode.

## Data Availability

The raw data that support the findings of this study are stored within the Institute for Applied Health Research Berlin GmbH (InGef, www.InGef.de). Restrictions apply to the availability of these data, and they are not publicly available/ cannot be shared, due to InGef data protection regulations. Access to patient-level data is not possible and all analyses must be conducted by InGef. Requests for bespoke analyses/ aggregate results are reviewed and approved by InGef.

## Abbreviations

AOM: Acute otitis media
CI: Confidence interval
CY: Child-years
EBM: Einheitlicher Bewertungsmaßstab—German uniform evaluation standard
ICD-10-GM: 10Th revision of the International Classification of Diseases, German Modification
InGef: Institute for Applied Health Research Berlin GmbH
IR: Incidence rates
OPS: Operationen und Prozedurenschlüssel—German procedure classification
PCV: Pneumococcal conjugated vaccine
PZN: Pharmazentralnummer – pharmaceutical central number
SHIs: Statutory health insurance providers
S. pneumoniae: Streptococcus pneumoniae
STIKO: German standing committee on vaccination
