# Supplementary material for: Incidence of acute otitis media in children < 16 years old in Germany during 2014–2019
Source: BMC Pediatr. 2022 Apr 13;22:204. doi: 10.1186/s12887-022-03270-w (PMC9006409; doi:10.1186/s12887-022-03270-w)
Supplement: Supplementary file 1 — Additional file 1: Supplementary Table 1. AOM and OME ICD-10-GM code definitions. Supplementary Table 2. EBM and OPS codes for estimation of diagnosis date. Supplementary Table 3. AOM-related complications code definitions. Supplementary Table 4. AOM-related surgical procedures code definitions. Supplementary Table 5. Incidence rates of AOM-related surgical procedures occurring during the same calendar year as an AOM episode. [file 12887_2022_3270_MOESM1_ESM.docx]

**Supplementary information**

**Code definitions**

Supplementary Table 1: AOM and OME ICD-10-GM code definitions

| **Categories** | **Diagnosis** | **ICD 10 GM codes** |
| --- | --- | --- |
| AOM | Suppurative and unspecified otitis media | H66 |
| AOM | Otitis media in diseases classified elsewhere | H67 |
| OME | Nonsuppurative otitis media | H65 |

Supplementary Table 2: EBM and OPS codes for estimation of diagnosis date

| EBM codes | EBM description | OPS codes | OPS description |
| --- | --- | --- | --- |
| 03335, 04335 | Otoscopy | 8-151.4 | Lumbar puncture |
| 09324, 20324 | Tympanometry | None | None |
| None | None | 3-800, 3-820 | Skull MRI |
| None | None | 3-200, 3-220 | Petrosal CT |
| None | None | 5-200 | Myringotomy |
| None | None | 5-200.4 | Myringotomy without drainage |
| None | None | 5- 200.5 | Myringotomy with drainage |
| None | None | 5-200.y | Myringotomy - N/a |

Supplementary Table 3: AOM-related complications code definitions

| *Diagnosis* | *ICD 10 GM Codes* |
| --- | --- |
| Perforation of tympanic membrane | H72 |
| Otorrhea | H92.1 |
| Ottorhagia | H92.2 |
| Acute mastoiditis | H70.0 |

Supplementary Table 4: AOM-related surgical procedures code definitions

| OPS codes | OPS description |
| --- | --- |
| 5-200 | Myringotomy (with or without ventilation tube insertion) |
| 5-201 | Tympanostomy tube removal |
| 5-202.2 | Exploratory tympanotomy |
| 5-202.5 | Tympanotomy with sealing of the round and / or oval window membrane |

**Additional tables**

Supplementary Table 5. Incidence rates of AOM-related surgical procedures occurring during the same calendar year as an AOM episode

|  | **Overall**  **(2014-2019)** | **2014** | **2015** | **2016** | **2017** | **2018** | **2019** | **Trend test**  **(p-value)*** |
| --- | --- | --- | --- | --- | --- | --- | --- | --- |
| **Overall (all age-groups)** |  |  |  |  |  |  |  |  |
| *N episodes* | 9,677 | 1,857 | 1,822 | 1,690 | 1,614 | 1,482 | 1,453 |  |
| *Rate per 1,000 CY*  *(95% CI)* | 40.87  (40.06-41.69) | 41.03  (39.19-42.94) | 41.64  (39.75-43.60) | 39.74  (37.87-41.68) | 42.01  (39.99-44.11) | 39.99  (37.98-42.08) | 41.40  (39.30-43.58) | 1.0 |
| **0-1 age group** |  |  |  |  |  |  |  |  |
| *N episodes* | 1,321 | 254 | 265 | 223 | 186 | 202 | 206 |  |
| *Rate per 1,000 CY*  *(95% CI)* | 27.35  (25.89-28.86) | 28.11  (24.76-31.78) | 30.78  (27.19-34.72) | 26.03  (22.73-29.68) | 23.59  (20.32-27.24) | 27.13  (23.52-31.14) | 29.33  (25.46-33.62) | 1.0 |
| **2-4 age group** |  |  |  |  |  |  |  |  |
| *N episodes* | 6,053 | 1,155 | 1,127 | 1,044 | 1,018 | 913 | 939 |  |
| *Rate per 1,000 CY*  *(95% CI)* | 62.74  (61.17-64.34) | 63.26  (59.66-67.02) | 64.82  (61.09-68.72) | 61.25  (57.59-65.08) | 64.19  (60.31-68.26) | 59.71  (55.90-63.71) | 62.03  (58.13-66.13) | 0.47 |
| **5-15 age group** |  |  |  |  |  |  |  |  |
| *N episodes* | 2,303 | 448 | 430 | 423 | 410 | 367 | 308 |  |
| *Rate per 1,000 CY*  *(95% CI)* | 25.04  (24.02-26.08) | 24.94  (22.69-27.36) | 24.21  (21.98-26.62) | 25.01  (22.68-27.51) | 27.94  (25.30-30.78) | 25.63  (23.07-28.39) | 23.81  (21.23-26.62) | 1.0 |

* Mann-Kendall test for trend
